# The Safety Profile of Common COVID-19 Vaccines in Patients With Multiple Sclerosis

**DOI:** 10.7759/cureus.54852

**Published:** 2024-02-25

**Authors:** Yasser S Aladdin, Danah A Alqarni, Sheifa W Alamoudi, Abdulrahman A Alharbi, Waad A Fudhah, Ghaida Alghamdi, Ahmed Attar

**Affiliations:** 1 Department of Neurology, Ministry of the National Guard-Health Affairs, Jeddah, SAU; 2 College of Medicine, King Saud Bin Abdulaziz University for Health Sciences, Jeddah, SAU; 3 Research, King Abdullah International Medical Research Center, Jeddah, SAU

**Keywords:** covi-19 vaccine, covid-19, disease-modifying therapies, side effect post vaccination, s: multiple sclerosis

## Abstract

Background and objective

Severe acute respiratory syndrome coronavirus 2 (SARS-CoV-2) is the causative agent of coronavirus disease 2019 (COVID-19). In light of the COVID-19 pandemic that emerged in late 2019, the World Health Organization (WHO) has endorsed mass immunization to enhance the population’s immunity against the virus. However, certain concerns have been raised about the safety of COVID-19 vaccines among patients with autoimmune disorders, including those with multiple sclerosis (MS). Further research is required to address these concerns and to gain deeper insights into the possible complications of COVID-19 vaccines among MS patients. This study aimed to assess the side effects of COVID-19 vaccines among MS patients.

Methods

An observational cross-sectional study was conducted between May and November 2023 at the National Guard Hospital, Jeddah, Saudi Arabia. All MS patients enrolled in our local registry system and provided phone numbers were included in the study. A total of 208 MS patients were surveyed via phone interviews, and data were collected regarding their demographics, MS history, COVID-19 history, SARS-CoV-2 vaccination status, and their exposure to disease-modifying therapies (DMTs). All results were analyzed using Stata software. Statistical significance was set at a CI of 95% and a p-value <0.05.

Results

In our cohort, 128 (61.5%) patients had received three doses of the COVID-19 vaccine, while 68 (32.7%) had received two doses; four patients (2.0%) had received only one dose, five (2.4%) had not received the vaccine, and the number of doses was unknown for the remaining three patients (1.4%). The BNT162b2 mRNA COVID-19 Vaccine from Pfizer-BioNTech was the most commonly administered (n=136 patients, 66.0%), followed byChAdOx1 nCoV-19 Vaccine from Oxford-AstraZeneca (n=47 patients, 22.8%), and mRNA-1273 SARS-CoV-2 Vaccine from Moderna (n=5 patients, 2.4%). Of note, 139 patients (69.5%) reported experiencing adverse events after receiving the vaccine, and the ChAdOx1 nCoV-19 Vaccine from Oxford-AstraZeneca was significantly associated with higher rates of side effects, in 87.8% of the patients.

Conclusion

A sizable proportion of MS patients experienced self-limiting side effects from exposure to the COVID-19 vaccine. The rates and incidence of side effects were similar to those encountered in the general population. None of the adverse effects recorded in our population of MS patients were serious or life-threatening. We recommend that physicians encourage patients with MS who have never received COVID-19 vaccination to get promptly vaccinated as the risks of COVID-19 infection far outweigh the minor risks associated with COVID-19 vaccination.

## Introduction

Coronavirus disease 2019 (COVID-19), caused by severe acute respiratory syndrome coronavirus 2 (SARS-CoV-2), is rapidly transmitted by nasal route through close contact with infected people. In January 2020, in the wake of the then-emerging COVID-19 pandemic, the World Health Organization (WHO) made a historical public health announcement to appeal for international cooperation to control the spread of the virus worldwide [1].

According to several studies, there is no specific treatment for COVID-19. Many therapeutic interventions have been employed to alleviate the symptoms of the disease and to reduce the severity of the complications. WHO has strongly promoted mass vaccination against the virus to strengthen the population's immunity. So far, more than 100 vaccines against COVID-19 have been introduced, some of which are still undergoing experimentation and testing. Although many of these vaccines are considered to be safe agents in general, they have also been linked to several adverse effects, including some fatal ones associated with events of thrombosis and anaphylaxis [1].

Two vaccines developed by AstraZeneca and Pfizer-BioNTech have been approved by the Saudi Ministry of Health to be used locally in the country. Even though the Ministry of Health has emphasized the importance and benefits of these vaccines, multiple concerns have been raised about the efficacy and safety of COVID-19 vaccines, leading to vaccine hesitancy among a significant segment of the population. Numerous factors could be related to the hesitancy regarding vaccines, including misconceptions, misleading information, and conspiracy theories [2].

Such misleading information has been found to be especially prevalent related to those suffering from neurodegenerative diseases. Since there have been reported cases of other previously introduced vaccines causing many issues, it became crucial to gain enough knowledge about whether the COVID-19 vaccine could cause substantial neurological complications. A study in the United States reported five case series involving the original onset of multiple sclerosis (MS) diagnosis after administering the mRNA COVID-19 vaccine [3]. Moreover, a 2002 study by Briggs et al. described the reactogenicity of MS after the administration of the vaccine, indicating that younger individuals, individuals with less physical damage, and individuals treated with disease-modifying therapies (DMTs) are at an increased risk for MS reactogenicity [4]. Concerns were raised about the impact of COVID-19 vaccination on patients suffering from MS.

MS is an autoimmune, demyelinating, neurodegenerative disease [5]. It is one of the most common inflammatory neurological disorders targeting the central nervous system (CNS), with an estimated prevalence of 40.40/per 100,000 population in Saudi Arabia in 2020. MS causes persistent impairment that can significantly affect productivity and quality of life among patients [6]. The causative agents of MS are still undefined and constitute a subject of active research [7]. However, multi-factorial environmental triggers, including infections, vitamin D deficiency, ultraviolet B (UVB) exposure, and specific genetic susceptibilities are believed to lead to MS [7]. The pathogenesis of the coronavirus causing the reactogenicity of MS is uncertain, although such viruses might activate self-reactive T cells attacking the CNS [3].

According to the recommendations of the National Multiple Sclerosis Society and other expert organizations, all patients with MS should get vaccinated against COVID-19 [8]. However, the data regarding the reactogenicity of COVID-19 vaccines for MS individuals is scarce, especially for those who are treated with DMTs, since it is unknown how DMTs affect immune response effectiveness [8]. Hence, further research is required to determine the potential complications among MS patients receiving the vaccine. Against this backdrop, this study aims to assess and determine the side effects of COVID-19 vaccines among patients with MS.

## Materials and methods

Study design and setting

This was a cross-sectional analysis conducted at the National Guard Hospital, in Jeddah, Saudi Arabia between May and November of 2023.

Study population

All MS patients with phone numbers enrolled in the best-care system were included. The study population was 448 and the survey was obtained and validated from previously published literature.

Sampling methodology

The sample size was calculated by using Raosoft software. The required sample size was estimated at a confidence level of 95%, a margin of error of 5%, and a 50% response distribution. The sample size was determined to be 208 participants, and the data were gathered from participants via phone interviews. MS patients were asked to answer questions about their demographics, MS history, COVID-19 history, SARS-CoV-2 vaccinations, and DMTs. MS history included the onset of diagnosis, subtype (relapsing-remitting, secondary progressive, primary progressive, and clinically isolated syndrome), patient-determined disease steps (PDDS), symptom screening, and any recent exacerbation or relapse. Moreover, the COVID-19 history included any previous infection of COVID-19, the date of infection, and if the patient was currently experiencing any COVID-19 symptoms. Also, patients were asked if they had faced any barriers regarding the administration of the COVID-19 vaccine and if they faced any severe reactions to other injections. Details about the COVID-19 vaccination were obtained by asking about the date, doses, type, and any kind of reactions the participants had experienced. Lastly, participants were asked to provide their recent DMT histories including dosage, any alteration in dosage due to COVID-19 vaccination, type of DMT, and the date of last DMT administration.

Data analysis

The data were refined and encoded on Microsoft Excel. The statistical analyses were conducted using Stata software (StataCorp. 2023, College Station, TX). Continuous variables were presented as means and standard deviations (SD), whereas categorical variables were presented as frequencies and percentages (%). Study subjects were compared based on their characteristics and the incidence of reactions or side effects after taking the COVID-19 vaccine by using the Chi-square test, assessing the association between relevant categorical variables. A cut-off p-value of <0.05 at a 95% confidence interval was considered statistically significant for all analyses performed.

## Results

Demographics and clinical features

A total of 208 patients who were clinically diagnosed with MS were included in this cross-sectional study. The mean age of patients was 37.1 years (SD: 9.6 years), with a female predominance (n=116, 55.8%). The patients’ mean height and weight were 163.8 cm (SD: 11.0 cm) and 72.6 kg (SD: 17.3 kg), respectively (Table 1). Among those patients, the mean duration of MS was estimated to be 9.5 years (SD: 5.2 years), with the relapsing-remitting type being the most common (n=72, 34.6%). Additionally, over half of the patients experienced an exacerbation or relapse of the disease (n=114, 54.8%) throughout their lifetime. Most of the patients (n=180, 86.5%) were currently on DMT (Table 1).

**Table 1:** Demographics and clinical characteristics of the cohort (N=208) SD: standard deviation; MS: multiple sclerosis; DMT: disease-modifying therapy for multiple sclerosis; COVID-19: coronavirus disease 2019

Variable	Variables
Mean age (SD), years	37.1 (9.6)
Female sex, n (%)	116 (55.8)
Mean height (SD), cm	163.8 (11.0)
Mean weight (SD), kg	72.6 (17.3)
Mean duration of MS (SD), years	9.5 (5.2)
Types of MS, (N=208), n (%)	
Relapsing-remitting	72 (34.6)
Secondary progressive	6 (2.9)
Clinically isolated syndrome	3 (1.4)
Radiologically isolated syndrome	1 (0.5)
Unknown	126 (60.6)
Previous MS exacerbation or relapse (N=208), n (%)	
Yes	114 (54.8)
No	91 (43.8)
Unknown	3 (1.4)
Current DMT, (N=208), n (%)	
Yes	180 (86.5)
No	27 (13.0)
Unknown	1 (0.5)
DMT within the past year (n=38), n (%)	
Yes	15 (39.5)
No	23 (60.5)
Tested positive for COVID-19 (N=208), n (%)	
Yes	82 (39.4)
No	123 (59.2)
Unknown	3 (1.4)
Current COVID-19-related symptoms (n=166), n (%)	
Yes	8 (4.8)
No	157 (94.6)
Unknown	1 (0.6)
Previous reactions or side effects to injections other than the COVID-19 vaccine, (n=203), n (%)	
Yes	35 (17.2)
No	151 (74.4)
Never had an injection	10 (5.0)
Unknown	7 (3.4)
Number of COVID-19 vaccine doses (N=208), n (%)	
0	5 (2.4)
1	4 (2.0)
2	68 (32.7)
3	128 (61.5)
Unknown	3 (1.4)
DMT dosing changes due to COVID-19 vaccine (N=193), n (%)	
Yes	7 (3.6)
No	174 (90.2)
Not applicable	10 (5.2)
Unknown	2 (1.0)
DMT taken on the day of COVID-19 vaccine (n=143), n (%)	
Gilenya (fingolimod)	15 (10.5)
Ocrevus (ocrelizumab)	11 (7.7)
Avonex (interferon beta-1a)	7 (4.9)
Tysabri (natalizumab)	3 (2.1)
Aubagio (teriflunomide)	2 (1.4)
Zeposia (ozanimod)	1 (0.7)
Tecfidera (dimethyl fumarate)	1 (0.7)
Other	28 (19.6)
Not taken on that day	75 (52.4)
Immunosuppressor or immuno-modifying drugs before COVID-19 vaccine (n=190), n (%)	
Ocrevus (ocrelizumab)	23 (12.0)
Aubagio (teriflunomide)	2 (1.1)
Avonex (interferon beta-1a)	2 (1.1)
Steroids	2 (1.1)
Gilenya (fingolimod)	1 (0.5)
Tysabri (natalizumab)	1 (0.5)
Other	16 (8.4)
Unknown	14 (7.4)
None	129 (67.9)

**Figure 1:**
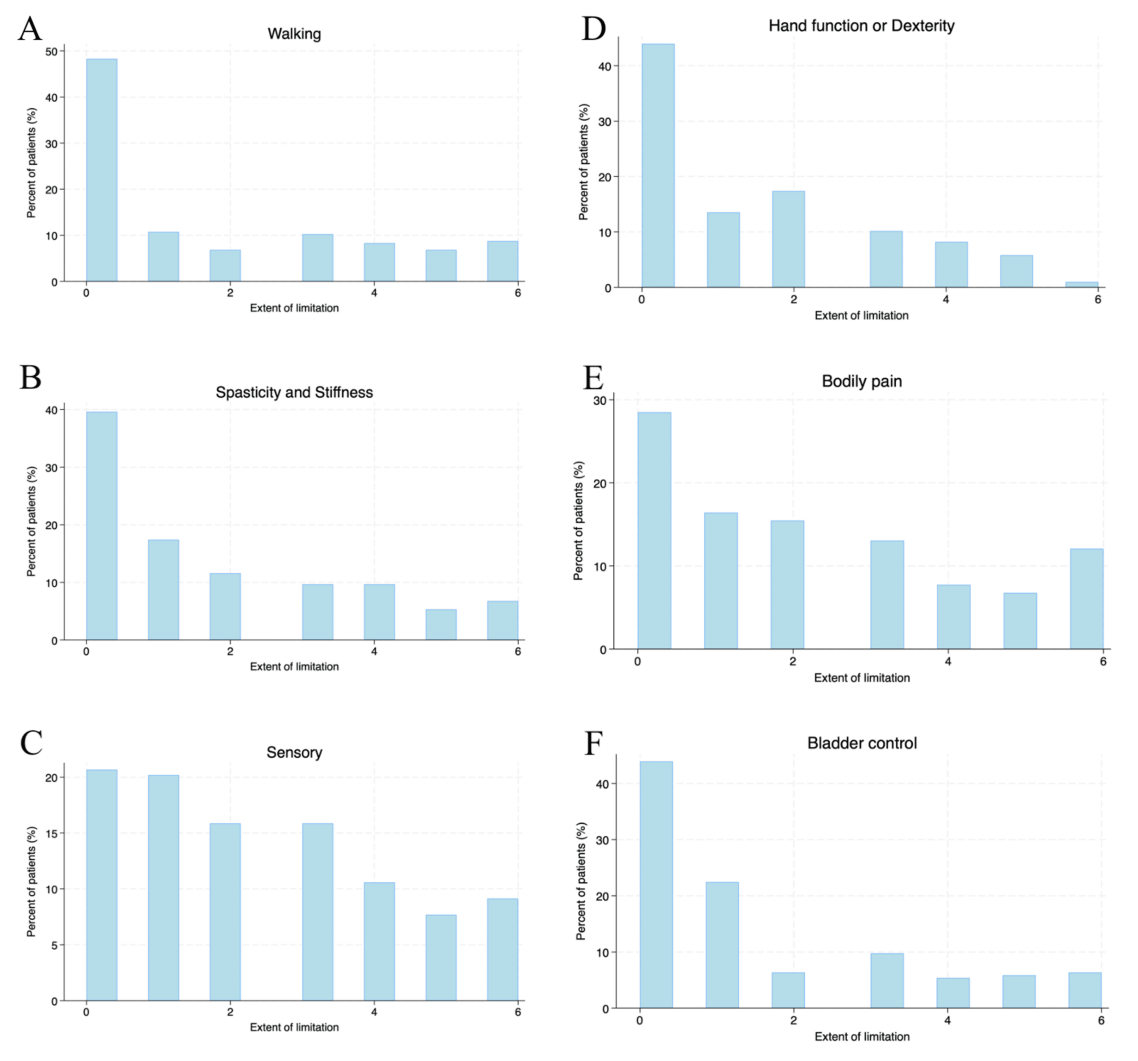
Histogram charts of various symptoms associated with multiple sclerosis - 1 The extent of limitation: 0: not affected at all; 1: very mild limitation; 2: mild limitation; 3: moderate limitation; 4: severe limitation; 5: very severe limitation; and 6: total limitation. The symptom analysis included (A) walking difficulties; B. spasticity and stiffness; C. sensory disturbances; D. hand function of dexterity; E. bodily pain; F. bladder control

**Figure 2:**
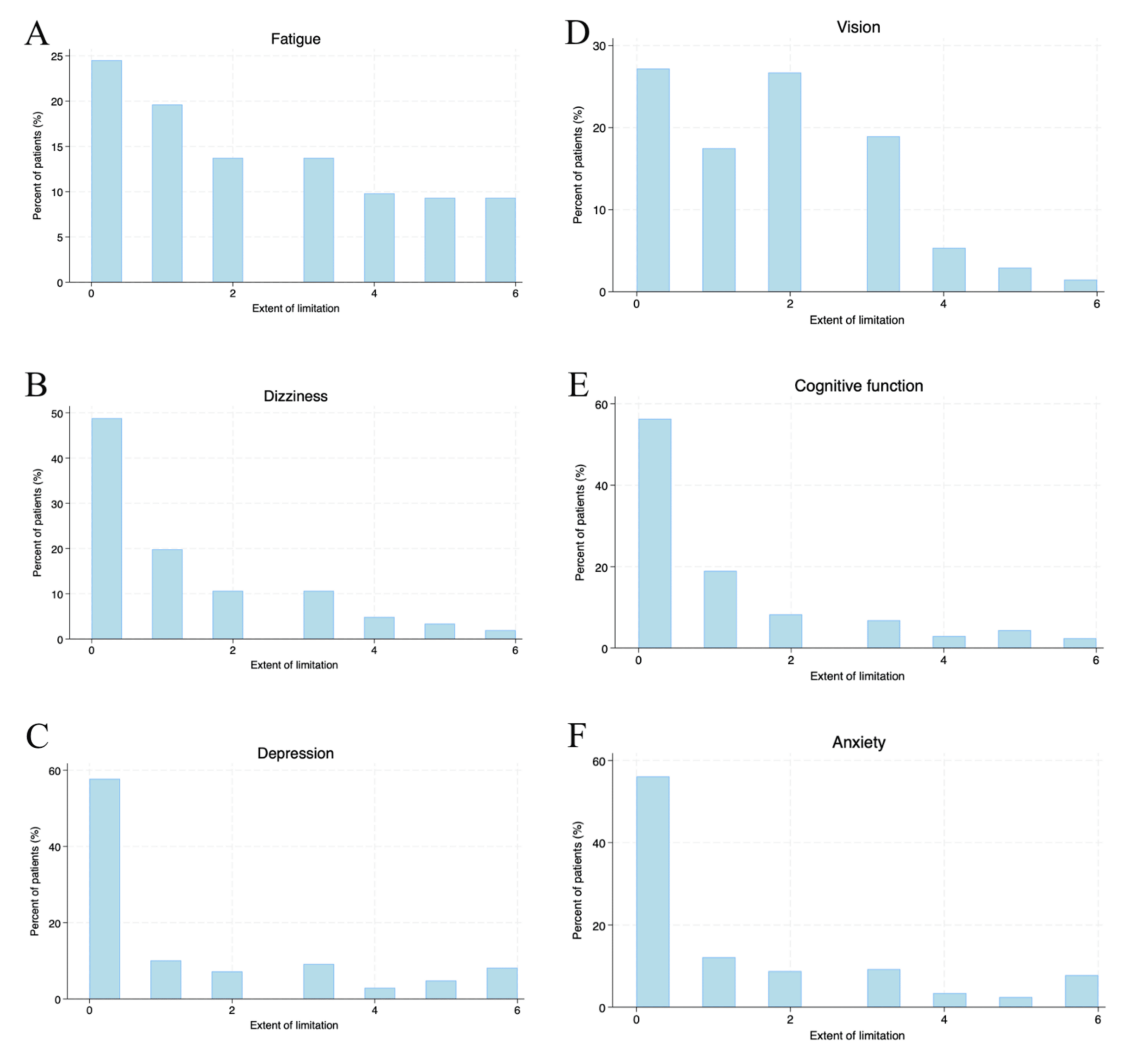
Histogram charts of various symptoms associated with multiple sclerosis - 2 The extent of limitation: 0: not affected at all; 1: very mild limitation; 2: mild limitation; 3: moderate limitation; 4: severe limitation; 5: very severe limitation; and 6: total limitation. The symptom analysis included (A) fatigue; (B) dizziness; (C) depression; (D) vision; (E) cognitive function; (F) anxiety

Clinical symptoms that interfered with everyday life activities, such as walking, hand function, stiffness, bodily pain, sensory function, bladder control, fatigue, vision, dizziness, cognitive function, depression, and anxiety were recorded based on the extent of limitation imposed. While “0” indicated “not affected at all”, “6” indicated total limitation of that activity, with degrees in between (“1” to “5”) indicating a range of limitation from mild to severe (Figures 1-2).

COVID-19 vaccine: characteristics and factors associated with side effects

A total of 82 (39.4%) patients were tested positive for COVID-19. However, only eight (4.8%) of them had recently experienced symptoms that they attributed to COVID-19. The majority of the patients (n=151, 74.4%) had never experienced previous reactions or side effects attributable to injections other than the COVID-19 vaccine in their lives (Table 2).

**Table 2:** Reactions or side effects after COVID-19 vaccination (n=452)* *This represents the number of reactions or side effects, not the patients (most of the patients experienced multiple reactions or side effects) COVID-19: coronavirus disease 2019

Variable	N (%)
Pain, soreness, or tenderness (injection site)	90 (20.0)
Fatigue	86 (19.0)
Fever or feeling feverish	85 (18.8)
Headache	73 (16.2)
Muscle aches (other than at the injection site)	23 (5.1)
Malaise (general feeling of discomfort, illness, or uneasiness)	19 (4.2)
Joint pain	14 (3.1)
Redness (injection site)	13 (2.9)
Chills	13 (2.9)
Swelling (injection site)	11 (2.4)
Warmth (injection site)	9 (2.0)
Itch (injection site)	9 (2.0)
Immediate allergic reaction (rash, swelling, difficulty breathing, fast heartbeat, dizziness, or fainting, etc.)	2 (0.4)
Menstrual cycle changes	2 (0.4)
Nausea	1 (0.2)
None	2 (0.4)

**Figure 3:**
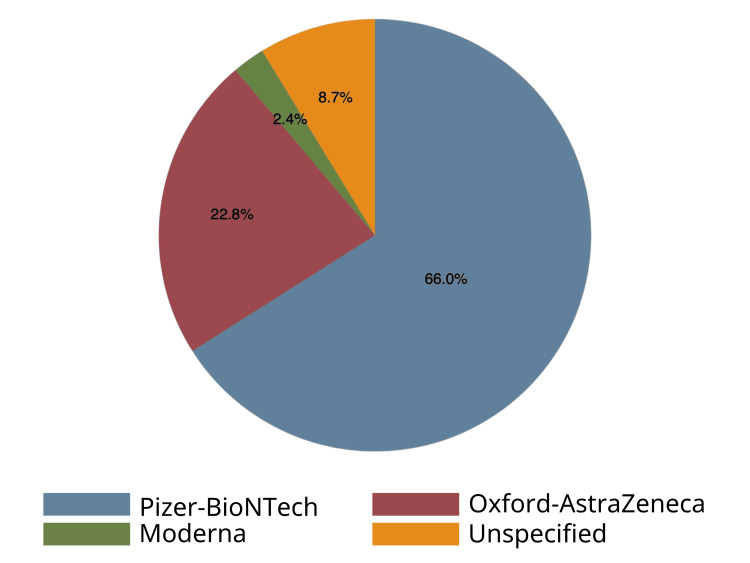
COVID-19 vaccination variants in patients with multiple sclerosis COVID-19: coronavirus disease 2019

Most of the patients (n=128, 61.5%) had received their third dose of COVID-19 vaccine (Table 1). The most commonly administered COVID-19 vaccine was manufactured by Pfizer-BioNTech (n=136, 66.0%), followed by Oxford-AstraZeneca (n=47, 22.8%), and Moderna (n=5, 2.4%) (Figure 3). Following vaccination, many reactions and side effects were experienced by the patients, such as pain, soreness, or tenderness (n=90, 20.0%), fatigue (n=86, 19.0%), fever or feeling feverish (n=85, 18.8%), and headache (n=73, 16.2%) (Table 2).

Further, a small group of patients (n=7, 3.6%) had to change their DMT dosing due to the COVID-19 vaccination, while the majority continued their treatment without any changes (n=174, 90.2%). The most commonly used DMTs on the day of receiving COVID-19 vaccine were Gilenya (fingolimod) (n=15, 10.5%), followed by Ocrevus (ocrelizumab) (n=11, 7.7%), and Avonex (interferon beta-1a) (n=7, 4.9%); of note, 75 patients (52.4%) did not take any DMT on the day of vaccination. Moreover, 67.9% (n=129) of the patients did not take any immunosuppressive or immune-modifying therapies before receiving the COVID-19 vaccine (Table 1).

Patients who experienced side effects after receiving the COVID-19 vaccine made up 69.5% (n=139) of the entire cohort. The vaccine manufactured by Oxford-AstraZeneca was significantly associated with a higher incidence of side effects (n=36, 87.8%) following the vaccine administration compared to the other vaccines (p=0.013; Table 3). Other factors, such as sex, type of multiple sclerosis, and number of COVID-19 vaccine doses were not found to be significantly associated with the incidence of side effects (Table 3).

**Table 3:** Comparison of patient characteristics based on the incidence of side effects after COVID-19 vaccination (n=200) *Association is significant at the 0.05 level (text in bold), based on the Chi-square test MS: multiple sclerosis

Variable	Side effects after COVID-19 vaccination, n (%)			
	Yes	No		P-value
No. of patients	139 (69.5)	61 (30.5)		
Sex				
Male	57 (64.0)	32 (36.0)		0.15
Female	81 (73.6)	29 (26.4)		
Type of MS				
Relapsing-remitting	39 (57.3)	29 (42.7)		0.16
Secondary progressive	3 (75.0)	1 (25.0)		
Clinically isolated syndrome	0 (0.0)	3 (100.0)		
Radiologically isolated syndrome	1 (100.0)	0 (0.0)		
Number of COVID-19 vaccine doses				
1	4 (100.0)	0 (0.0)		0.33
2	48 (72.7)	18 (27.3)		
3	87 (68.0)	41 (32.0)		
COVID-19 vaccine type				
Pfizer-BioNTech	83 (63.9)	47 (36.2)		0.013*
Oxford-AstraZeneca	36 (87.8)		5 (12.2)	
Moderna	4 (80.0)		1 (20.0)	

## Discussion

The National Multiple Sclerosis Society has released recommendations endorsing COVID-19 vaccination for all MS patients regardless of the type, severity, or disease activity. Our observational study comprehensively collected and assessed detailed demographic and clinical parameters, including age, gender, disease duration, relapse activity, vaccine type, and prevailing DMT usage among MS patients.

However, the data on the reactogenicity of COVID-19 vaccines in MS patients on various DMTs are limited. The precise biological interactions between COVID-19 vaccines and each DMT need to be elucidated, including the effectiveness of various COVID-19 vaccines in patients actively receiving DMTs. The objective of our study was to evaluate the adverse effects of COVID-19 vaccines and to assess the safety profile of each COVID-19 vaccine in patients with MS on various DMTs. The clinical and demographic characteristics of our patient group align with the typical profile of MS patients worldwide. Our cross-sectional study included 208 MS patients with a mean age of 37.1 years, predominantly female, a mean disease duration of 9.5 years, and with relapsing-remitting type of MS being the most prevalent subtype. More than half of the patients had experienced disease exacerbations and the majority were actively receiving immunomodulatory therapy with various DMTs (86.5%).

More than 60% of our patient population had never tested positive for COVID-19. A significant majority (74.4%) had no history of reactions to any injection other than the COVID-19 vaccine. However, a significant proportion (69.5%) of patients had experienced side effects following COVID-19 vaccination. Various reactions were reported, including injection site pain, fatigue, fever, and headache. The ChAdOx1 nCoV-19 Vaccine was associated with a significantly higher incidence of reported side effects compared to other vaccines (87.8%). Other factors, including sex, MS subtype, and number of vaccine doses did not show any significant association with the rates of side effects.

False-negative SARS-CoV-2 antibody tests have been reported in two MS patients on ocrelizumab who contracted COVID-19 [9]. The theoretical risk of blunting production of SARS-CoV-2 antibodies exists as a substantial portion of our patients reported no prior positive COVID-19 tests despite experiencing typical COVID-19 symptoms at some stage of their disease. An alternative consideration is the altered accuracy of COVID-19 testing in MS patients with the potential for false-negative results in RT-PCR tests [10]. Within our MS patient population, the predominant side effects were injection site pain, fatigue, fever, and headache. This is consistent with the side effects previously observed in the general population following COVID-19 vaccination [11]. One large study involving 718 patients within the iConquerMS cohort reported that more than 64% of MS patients had encountered vaccine reactions within the initial 24 hours with a higher prevalence in patients receiving ChAdOx1 nCoV-19 vaccine compared to those who received BNT162b2 mRNA COVID-19 Vaccine and mRNA-1273 SARS-CoV-2 Vaccine [4].

Our study revealed an incidence of adverse effects of 69.5% among our MS patients, with ChAdOx1 nCoV-19 Vaccine being most frequently associated with such events. In a recent investigation, over 20% of MS patients reported concerns about the safety, efficacy, and side effect profile of COVID-19 [12,13]. Among those patients, 26% had major concerns about the short-term side effects, while 49% regarded them as minor concerns [13,14]. Our study demonstrates the relative safety and efficacy of COVID-19 vaccines in MS patients with comparable rates of adverse reactions to the general population, irrespective of the utilization of commonly used DMTs for MS patients. Our study may provide another analysis of the assessment of benefits against potential minor risks of COVID-19 vaccines in MS patients who are actively using DMTs. The findings might be validated by conducting prospective multicenter studies to assess the longstanding sequelae of COVID-19 vaccines in MS patients. 

Serious, but rarer, side effects have been described following the administration of various COVID-19 vaccines, including reports of anaphylaxis, myocarditis, pericarditis, venous sinus thrombosis, ischemic strokes, new-onset refractory status epilepticus (NORSE), thrombosis with thrombocytopenia syndrome (TTS), and Guillain-Barré syndrome (GBS). The Centers for Disease Control and Prevention has stated that the benefits of the mRNA COVID-19 vaccine outweigh the risks of side effects [15-17].

This study provides a comprehensive assessment relating to various demographic and clinical parameters such as age, gender, disease duration, relapse history, and prevailing disease-modifying therapy (DMT) usage. Furthermore, it unveils factors related to potential side effects after COVID-19 vaccination, including vaccine type. However, this study has a few limitations, especially in terms of gathering data, due to the data being based on phone-call questionnaires, which may entail the potential for respondent bias. Moreover, the study sample was confined to patients from the Western region of the country, which may limit the generalizability of our findings to the broader population. Finally, our assessment only considers the short-term and intermediate-term side effects in this population, and another evaluation of the same cohort may be required to assess the long-term effects.

## Conclusions

The safety profile of COVID-19 vaccines in patients with MS is comparable to the general population with similar rates of side effects. A sizable segment of patients with MS experienced untoward effects after receiving the COVID-19 vaccine regardless of sex, MS type, and number of COVID-19 vaccine doses. The side effects are generally minor and self-limiting without any serious or life-threatening reactions. The ChAdOx1 nCoV-19 Vaccine (from Oxford-AstraZeneca) vaccine was significantly associated with higher rates of side effects compared to BNT162b2 mRNA COVID-19 Vaccine (from Pfizer-BioNTech) and mRNA-1273 SARS-CoV-2 Vaccine (from Moderna). Patients with MS who have never received the COVID-19 vaccine due to misconceptions about vaccine safety should be encouraged to complete their COVID-19 vaccination as the benefits of the COVID-19 vaccine may outweigh the risk of common, but minor and transient adverse reactions that are comparable to those among the general population.
