# Correction: The Safety Profile of Common COVID-19 Vaccines in Patients With Multiple Sclerosis

**DOI:** 10.7759/cureus.c390

**Published:** 2026-01-07

**Authors:** Yasser S Aladdin, Danah A Alqarni, Sheifa W Alamoudi, Abdulrahman A Alharbi, Waad A Fudhah, Ghaida Alghamdi, Ahmed Attar

**Affiliations:** 1 Department of Neurology, Ministry of the National Guard-Health Affairs, Jeddah, SAU; 2 College of Medicine, King Saud Bin Abdulaziz University for Health Sciences, Jeddah, SAU; 3 Research, King Abdullah International Medical Research Center, Jeddah, SAU

This article has been updated to correct the affiliations for Yasser Aladdin and Ahmed Attar.

